# Supplementary material for: Splice-Junction-Based Mapping of Alternative Isoforms in the Human Proteome
Source: Cell Rep. Author manuscript; Available in PMC 2020 Jan 15. (PMC6961840; doi:10.1016/j.celrep.2019.11.026)
Supplement: 2 [file NIHMS1546469-supplement-2.zip › DF1/PXD009737/TestisTrypsin/TestisTrypsin_19_SRP14_AAAAAAAAAPAAAATAPTTAATTAATAAP.pdf]

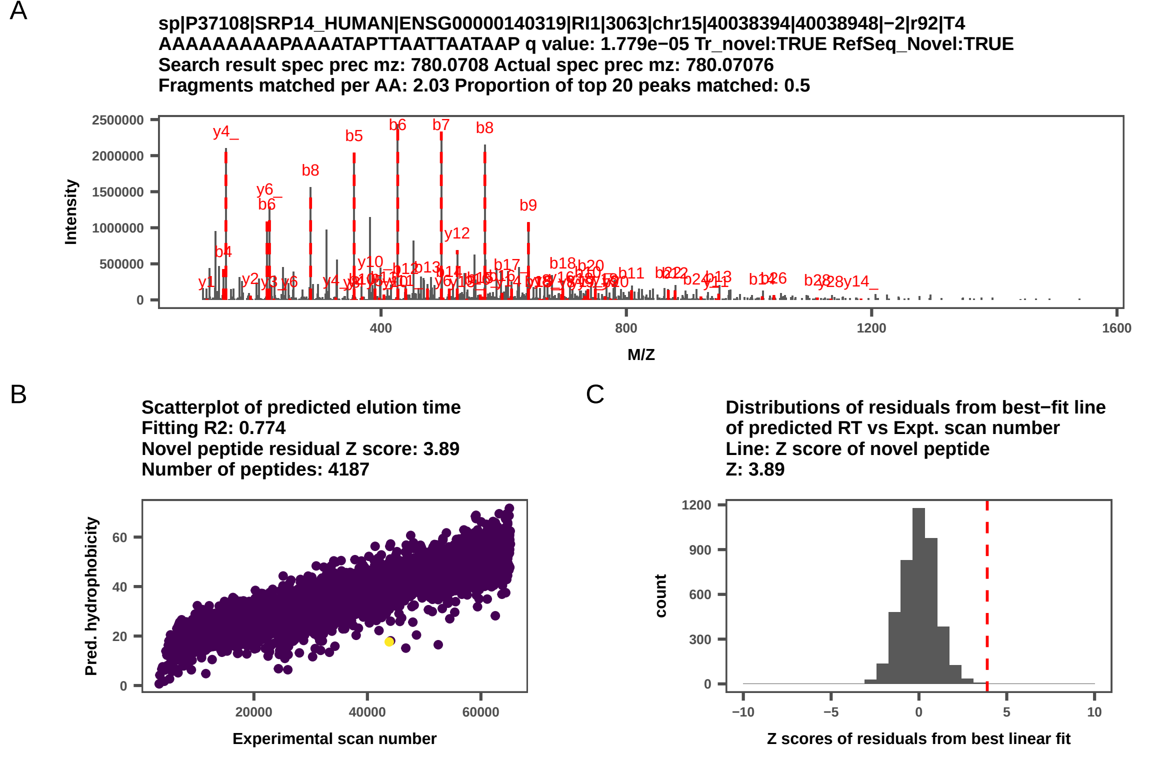
A
sp|P37108|SRP14_HUMAN|ENSG00000140319|RI1|3063|chr15|40038394|40038948|−2|r92|T4
AAAAAAAAAPAAAATAPTTAATTAATAAP q value: 1.779e−05 Tr_novel:TRUE RefSeq_Novel:TRUE
Search result spec prec mz: 780.0708 Actual spec prec mz: 780.07076
Fragments matched per AA: 2.03 Proportion of top 20 peaks matched: 0.5
Intensity
2500000
2000000
1500000
1000000
500000
0
y4_
b5
b6
b7
b8
b8
y6_
b6
b9
y12
b4
y10_
b17
b18
b20
b11
y1
y2
y14_
400
800
1200
1600
M/Z
B
Scatterplot of predicted elution time
Fitting R2: 0.774
Novel peptide residual Z score: 3.89
Number of peptides: 4187
Pred. hydrophobicity
60
40
20
0
20000
40000
60000
Experimental scan number
C
Distributions of residuals from best−fit line
of predicted RT vs Expt. scan number
Line: Z score of novel peptide
Z: 3.89
count
1200
900
600
300
0
−10
−5
0
5
10
Z scores of residuals from best linear fit
